# Advances in Neuropsychology: Top Papers Published in *Brain Sciences* in 2022–2023

**DOI:** 10.3390/brainsci14060588

**Published:** 2024-06-09

**Authors:** Pierluigi Zoccolotti

**Affiliations:** 1 Department of Psychology, Sapienza University of Rome, 00185 Rome, Italy; pierluigi.zoccolotti@uniroma1.it; 2 Tuscany Rehabilitation Clinic, 52025 Montevarchi, Italy

The spectrum of typical neuropsychology topics has gradually broadened in recent years thanks to advances in neuroimaging and electrophysiological techniques. Thus, there has been a significant increase in contributions related on the one hand to understanding the brain correlates of social and emotional processes [1,2] and on the other focused on different types of pathologies characterized by stress [3], phobias [4], and various types of substance use disorders and addiction [5]. The most cited papers published in 2022–2023 in the “Neuropsychology” section reflect both this shift and the evolution of neuropsychological issues. Thus, a substantial number of studies have focused on social cognition and its potential risks, along with eating disorders, stress control, and psychotic episodes.

A typical example of this new approach is the review presented by Wadsley and Ihssen [6]. These authors focused on a major problem in today’s world, i.e., the use of social networking sites such as Facebook. In their review, they were able to trace enough relevant papers that adopted different MRI methodologies, including structural, resting-state, and task-related activation studies. Several significant findings emerged from this analysis that confirm the importance of focusing on this topic. For example, in structural MRI studies, a negative correlation was observed between the grey matter volume of critical areas (such as the nucleus accumbens, a part of the ventral striatum, the anterior cingulate cortex, and the amygdala) and excessive use of social networking sites. Evidence still does not allow us to draw causal conclusions, i.e., that structural abnormalities directly depend on the frequent/addictive use of social networking sites. However, longitudinal studies published in the last few years indicate the possibility of providing clarifying information on this issue. The overall conclusion from this review is that the excessive or problematic use of social networking sites has “some important neural similarities to substance abuse”, raising concerns about the importance of interventions to control and rationalize the use of websites.

In the realm of addictions, one relevant issue concerns the increasing emergence of new psychoactive substances which come on the market. Crucial information about these substances that is typically lacking in the early periods of use regards their potential toxic effects. The systematic review by de Oliveira et al. [7] focuses on synthetic cannabinoids. Originally synthesized for research purposes, these authors found that their main use was as drugs of abuse mixed with other various substances and herbs. Synthetic cannabinoids represent a large class of substances that have been developed in successive waves since the 1970s. This heterogeneity increases the complexity of analyzing these substances and makes it difficult to reach a unified assessment of their toxicity. The careful analysis carried out by the authors uncovered several toxic effects of synthetic cannabinoids, the most frequent of which were tachycardia and seizures, but also system disorientation, derealization, depersonalization, amnesia, and several others. Also, this analysis allowed them to identify the toxicity effects as a function of the specific type of substances; in particular, AB-CHMINACA and ADB-FUBINACA were the most dangerous varieties and caused the greatest frequency of symptoms and a substantial proportion of fatalities. Advanced knowledge of the consequences of synthetic cannabinoids is relevant for clinicians, particularly because their use is particularly risky in individuals with epilepsy and schizophrenia because of their potential capacity to trigger convulsive crises and obnubilation of consciousness.

Celeghin et al. [8] present a systematic and comprehensive review of the brain correlates of eating disorders, including anorexia nervosa, bulimia nervosa, and binge eating disorder. The literature on this area has grown considerably in recent years, and thus they were able to trace 30 informative studies. The three disorders show different clinical manifestations and symptoms and distinct neurofunctional correlates. Thus, anorexia nervosa is associated with hyperactivity in the amygdala, insula, and hypothalamus, bulimia nervosa is associated with hyperactivity of the insula and striatum, and binge eating is associated with activity in the temporal cortex and striatum. Understanding the neural correlates of eating disorders could significantly contribute to improvement in the treatment of these conditions.

Driving is a behavior that leads to numerous stressful situations associated with both task-related and situational components. Sciaraffa et al. [9] developed and validated a new stress measure (Neurometric) that can be used outside the laboratory to obtain information about stress levels in daily life situations. Neurometric features include a lightweight EEG that relies on two wet sensors in real-time and requires no calibration. In an experimental test of this new instrument, the authors show that Neurometric provided reliable estimates of stress levels compared to a standard measure (skin conductance level). They first tested the measure in a controlled multitasking condition in the laboratory and then in a driving task featuring realistic conditions, such as the requirement of fast driving and the presence of high traffic noise. Neurometric proved successful in discriminating among different levels of stress both in the multitasking session and the driving task. The authors advocate for using EEG measures rather than the isolated use of autonomic measures (which can be influenced by various external and difficult-to-control factors).

Gentsch and Kuehn [10] focus on how we store and retrieve bodily experiences of the past and how they might influence our everyday life. They trace the knowledge of the meaning and effect of body memories from the early psychoanalytic analysis of the repression of distressful memories and the neurological examination of patients with “asymbolia for pain” or patients with hippocampal damage. In their opinion paper, they present the “hypothesis that the storage and retrieval of bodily experiences offers a critical route to understand and modify mental health problems, in particular bodily trauma, psychosomatic and chronic pain, dissociative symptoms and general somatic symptoms”. This hypothesis has its foundation in the large body of literature on clinical symptoms and their potential origin in corporeal memories. In this vein, the authors examine in great depth the literature on trauma memories, psychosomatic and chronic pain, and clinical forms of somatoform dissociation, from daydreaming to depersonalization disorders. Overall, they propose that negative bodily experiences of the past might contribute to the development of somatic manifestations of mental health problems, even though they also emphasize that psychosomatic complaints are characteristically the result of a variety of biological, psychological, and sociocultural factors, with body memories only one of the concurring factors. While the focus of Gentsch and Kuehn’s [10] analysis is to stimulate research on body memories to understand and treat mental health conditions, they also offer initial suggestions on how interventions, such as training in bodily and somatosensory awareness, could be directed towards modifying dysfunctional body memories.

Tschentscher et al. [11] present an extensive analysis of cognitive impairments in patients with psychotic episodes. Based on the previous literature, the authors anticipate the idea that in their first episode, patients would suffer from a derangement of cognitive skills spanning across multiple areas (“global deficit hypothesis”). Previous evidence regarding the chronic stage is more limited but seems to indicate that cognitive deficits will persist over time without significant further deterioration (“longitudinal stability hypothesis”). In general, both hypotheses were supported based on the substantial number of relevant papers identified. Thus, patients with first-episode psychotic disorders showed severe deficits in multiple cognitive areas such as executive functioning, memory, working memory, psychomotor speed, and attention. Furthermore, there was limited evidence of a worsening of cognitive deficits in the chronic stage, which indicates a stability that is independent of the presence of antipsychotic medications. Fewer studies were available to evaluate the possible presence of cognitive deficits in patients at risk of psychotic episodes. This evidence appears heterogeneous and does not point to a profile of impairment at the high-risk stage, although more studies are needed to clarify this point. The presence of a widespread cognitive impairment underscores the importance of a thorough assessment spanning all critical cognitive areas being carried out before the start of the clinical treatment. 

Few events have changed our lives as dramatically as the recent COVID-19 pandemic. Discussion of the possible consequences of viral infection has involved the general population as never before. Ercoli et al. [12] published an extensive review on the impact of the pandemic on clinical and research activities, the use of telemedicine, the delivery of education and the psychological status of residents. Despite the short time range, they were able to trace twelve papers with relevant information. Their analysis offers interesting insights into the formative complexities associated with the pandemic.

A different line of research in the neuropsychological realm concerns the study of normal and atypical development over the life cycle. For example, studies in this area examine the changes that occur from a cognitive perspective in normal and pathological aging. In this context, a particularly rich and active area of research is the study of developmental learning disorders. The past year has seen significant contributions in these areas.

Cepukaityte et al. [13] carried out two experiments to examine the relationship between short- and long-term memory and its possible changes across the life span. The first experiment was instrumental in developing a new paradigm that would allow for comparing short- and long-term memories for the same contextual-spatial associations. The new continuous-report task was used to examine cross-sectional differences among young, middle-aged, and older adults. Short- and long-term memories were significantly related and impaired in older adults. A second experiment (carried out online) replicated and expanded these findings and introduced several methodological improvements. The authors frame the shared vulnerability in memory decay for short- and long-term memories with aging in relationship with the functionality of the medial temporal lobe and the hippocampus. Future studies are needed to confirm and substantiate these relationships and to relate these observations on healthy aging to disorders characterized by memory deficits, such as Alzheimer’s disease.

Finally, two papers concern the area of developmental dyslexia. Both are interested in understanding this learning disorder beyond the traditional interpretation of a phonological core deficit. Smith-Spark and Gordon [14] examined the large body of literature on children and adults with developmental dyslexia and found a variety of difficulties spanning from working memory to a variety of executive functions including planning, inhibition, updating, and set-shifting. Interestingly, various studies show that these difficulties extend to everyday life conditions and significantly affect the lives of children and adults with dyslexia. The authors note that a strictly phonological interpretation does not account for the wealth of accumulated evidence. For example, they note that children with dyslexia are impaired in visuospatial short-term memory tasks, not only verbal ones. The authors posit that the Model of the Control of Action may represent an effective framework to interpret the broad spectrum of cognitive difficulties associated with dyslexia. More research is certainly needed to understand the possible overlap of this complex pattern of findings in dyslexia with other neurodevelopmental conditions, such as attention deficit hyperactivity and autism spectrum disorders. In particular, the authors note that these conditions share some of the neuroanatomical substrates (i.e., cerebellar, and prefrontal cortex) discussed in the context of an executive interpretation of dyslexia. Future studies may help to uncover the possible role of comorbidities in the genesis of the executive deficits described.

Also, Premeti et al. [15] begin their review from the perspective of their interest in contrasting the phonological interpretation of dyslexia with an alternative theoretical view, i.e., the idea that one deficit at the base of the learning disorder is a reduced visual attention span that limits the number of letters processed in parallel. They extensively review the literature on event-related brain potentials and eye movement recordings. The data on event-related brain potentials indicate that children and adults with dyslexia show deficits at different processing stages. Interestingly, the authors note that these deficits can be found across languages with different levels of orthographic depth. The authors also review the large body of evidence on eye movements, highlighting the areas in which controversies still persist, particularly whether children with dyslexia show deficits in non-linguistic tasks. They propose the working hypothesis that the simultaneous recording of these two measures may provide insights into the multidimensional causes of dyslexia. In this vein, the authors quote two recent studies that adopted this methodology, although not with the aim of examining deficits in dyslexia. Thus, this proposal stands as an interesting challenge for future research.
